# Genetically predicted TTK inhibition and its association with reduced breast cancer risk: a two-step Mendelian randomization study of potential gut microbiome mediation

**DOI:** 10.1016/j.clinsp.2026.100961

**Published:** 2026-05-01

**Authors:** Jia Liu, Mohan Liu, Ying Xu, Zihao Wang, Xingtong Zhou, Qiang Sun

**Affiliations:** Department of Breast Surgery, Peking Union Medical College Hospital, Peking Union Medical College, Chinese Academy of Medical Sciences, Beijing, China

**Keywords:** TTK inhibition, Breast cancer, Gut microbiota, Mendelian randomization, Drug target MR, Mediation analysis, Causality

## Abstract

**Background:**

BOS172722, a selective TTK inhibitor, shows promise for Breast Cancer (BC) treatment. The role of gut microbiota as a potential mediator in this process has not been established. This Mendelian Randomization (MR) study investigated the causal effect of TTK inhibition on BC and conducted an exploratory analysis of potential mediation by gut microbiota.

**Methods:**

A two-step, two-sample drug target MR analysis used IEU Open GWAS project datasets. The inverse-variance-weighted method estimated causal effects, with sensitivity tests confirming robustness. False Discovery Rate (FDR) correction was applied to analyses for all microbial taxa.

**Results:**

Genetically predicted TTK inhibition significantly reduced BC risk (OR = 0.667, 95 % CI 0.543–0.819; FDR-adjusted *p* < 0.001). In secondary analyses, initial uncorrected results indicated nominal associations between TTK inhibition and 20 gut microbial taxa, and between 6 taxa and BC risk. However, none of these microbiota-related associations remained statistically significant after FDR correction (all q-values >0.1). An exploratory mediation analysis on *Genus Anaerostipes* id.1991, which showed the strongest nominal signals (TTK inhibition on *Anaerostipes*: uncorrected *p* = 0.003; *Anaerostipes* on BC: uncorrected *p* = 0.025), was performed for hypothesis generation only; no statistically significant mediation was observed.

**Conclusion:**

This study provides robust genetic evidence that TTK inhibition is causally associated with a decreased risk of breast cancer. The exploratory analysis of gut microbiota as a mediator did not yield statistically significant results after correction for multiple testing. The potential role of specific microbes, such as Genus Anaerostipes, remains inconclusive and requires further dedicated investigation.

## Highlights

• Genetically predicted TTK inhibition is causally associated with a significant decrease in breast cancer risk.
• No gut microbial taxa remained significantly associated with TTK inhibition or breast cancer after FDR correction.
• An exploratory mediation analysis of Genus *Anaerostipes* did not yield statistically significant results.
• The findings reinforce TTK as a promising therapeutic target for breast cancer.

## Introduction

Breast Cancer (BC) remains a significant global public health concern due to its high mortality.^1^, ^2^, ^3^ Since 2019, there has been a notable increase in the global incidence of BC,^4^ which now ranks second in global cancer incidence and fourth in mortality.^5^ Despite improvements in overall treatment modalities that have somewhat optimized oncological outcomes for BC patients, highly aggressive and less common subtypes of BC continue to present with elevated recurrence and mortality rates.^6^, ^7^, ^8^ Given this therapeutic challenge, developing new targeted therapeutic agents for BC patients is essential to improve their survival and prognosis.

Elevated expression of spindle assembly checkpoint kinase, Monopolar Spindle 1 (MPS1, also known as TTK), ^9^ is associated with genes critical to breast tumor growth and high histologic grade in BC.^10^^,^^11^ Consequently, TTK inhibitors are emerging as a promising avenue for targeted therapy of highly aggressive BC. Existing TTK inhibitors have demonstrated the ability to induce BC cell death and enhance sensitivity to chemotherapy.^12^^,^^13^ Previous studies have shown that the clinical candidate BOS172722, an inhibitor of TTK, significantly induces lethal sensitivity in Triple-Negative Breast Cancer (TNBC) cell lines and promotes tumor regression when combined with paclitaxel.^14^ However, a direct mechanistic link between TTK inhibition, specific gut microbes, and therapeutic outcomes in BC has not been established.

The gut microbiome, a relatively modifiable factor, has garnered increasing attention in BC research.^15^, ^16^, ^17^ Alterations in specific gut microbiota may contribute to BC risk and prognosis^18^^,^^19^ and impact clinicopathologic features such as Estrogen Receptor (ER) levels and tumor grade.^20^^,^^21^ Gut microbiota can also influence mechanisms of chemotherapeutic sensitization and interfere with tumor cell cycle regulation.^19^^,^^22^^,^^23^ Given that previous research on BOS172722 utilized oral administration, the authors propose a novel but untested hypothesis that gut microbiota may play a role in its therapeutic effect on BC. Some microbial metabolites, such as Short-Chain Fatty Acids (SCFAs) produced by genera like *Anaerostipes*,^24^ exert anti-inflammatory and anti-proliferative actions in various cancers,^25^^,^^26^ suggesting a plausible biological pathway.

Mendelian Randomization (MR) is a genetic epidemiology method that uses genetic variants as instrumental variables to infer causal relationships, minimizing confounding and reverse causation.^27^^,^^28^ Drug target MR, in particular, uses genetic proxies for drug action to evaluate therapeutic effects^29^^,^^30^ and has helped identify targets for various cancers.^31^, ^32^, ^33^ To our knowledge, no MR study has reported the association between TTK inhibitors and BC.

To address this knowledge gap, this study primarily aims to investigate the causal effect of genetically predicted TTK inhibition on BC risk. As a secondary, exploratory objective, the authors investigate a potential causal chain where TTK inhibition alters gut microbiota composition, which in turn impacts BC risk, to generate hypotheses for future research.

## Methods

### Study design

**Fig. 1:**
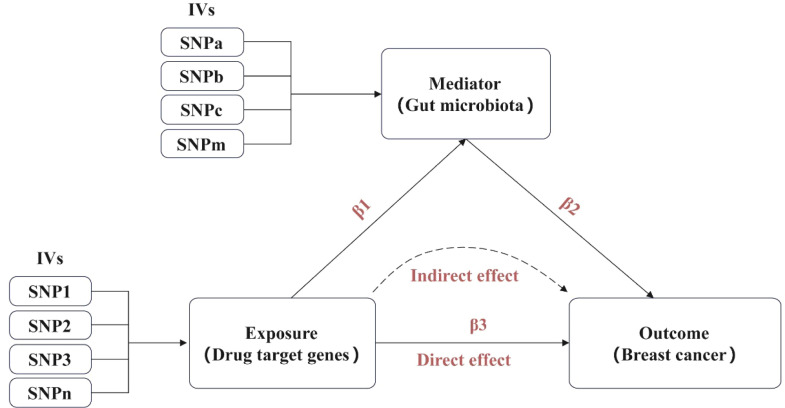
Overview of study design. β1 represents the Mendelian Randomization (MR) effect of TTK inhibition on gut microbiota, β2 the MR effect of gut microbiota on Breast Cancer (BC), and β3 the total MR effect of TTK inhibition on BC.

This study employed MR for a primary causal analysis and a secondary mediation analysis, as outlined in Fig. 1. The authors adhered to the Strengthening the Reporting of Observational Studies in Epidemiology-Mendelian Randomisation (STROBE-MR) guidelines.^34^ The MR approach relies on four key assumptions:.^35^1) Instrumental variables are strongly correlated with the exposure (F statistic >10). 2) Instrumental variables are independent of confounders. 3) Instrumental variables do not directly affect the outcome except through the exposure. 4) For drug-target MR, instrumental variables are located within cis-eQTLs (±500 kb) of the drug target gene.

### GWAS summary data sources

Genetic association estimates were obtained from the IEU Open GWAS Project (https://gwas.mrcieu.ac.uk/)..^36^ All participants were of European ancestry. Detailed dataset information is in Supplementary Table S1. The TTK eQTLs dataset comprised 31,684 samples. The BC dataset included 240,341 controls and 17,389 cases. The gut microbiota dataset had a sample size of 14,306.

### Filtering of instrumental variables

#### Selection of genetic instruments for drug target eQTLs

SNPs were selected from cis-expression Quantitative Trait Loci (eQTLs) within ±500 kb of the TTK gene. The authors chose genetic variants associated with TTK mRNA expression at *p* < 1 × 10^−5^ and Minor Allele Frequency (MAF) < 5 %. Variants were clumped to a Linkage Disequilibrium (LD) threshold of *r*² 〈 0.30 and a distance of 100 kb. Palindromic SNPs and those with MAF < 0.42 were excluded.

#### Selection of genetic instruments for gut microbiota

SNPs associated with 211 gut microbiota taxa were obtained. The authors identified SNPs reaching genome-wide significance (*p* < 1 × 10^−5^) and clumped them at *r*^2^ < 0.001 and a 10,000 kb window. Palindromic SNPs and those with MAF < 0.42 were excluded.

### Primary analysis

A two-sample MR analysis was performed to assess the causality between BOS172722 and BC. The primary method was the Inverse-Variance Weighted (IVW) model.^37^^,^^38^ Given the use of two SNPs, a fixed-effects model was employed. Supplementary analyses included weighted median, weighted mode, and simple mode.

#### Exploratory mediation analysis of gut microbiota

A two-step MR approach was used to explore potential mediation. First, the authors examined the effect of TTK inhibition on gut microbiota (β1). Second, the authors explored the effect of microbiota on BC (β2). The mediation proportion was calculated as (β1×β2) / β3, where β3 is the total effect of TTK inhibition on BC. The RMediation package provided 95 % Confidence Intervals (95 % CIs).

#### Sensitivity analysis

The authors used the PhenoScanner database to screen for pleiotropic associations of the instrumental variables with potential confounders.^39^^,^^40^ Heterogeneity was assessed using Cochran’s *Q* test and the I² statistic.^41^ The MR-Egger intercept and MR-PRESSO methods were used to detect directional pleiotropy where applicable.^42^^,^^43^ Leave-one-out analysis was conducted to assess the influence of individual SNPs.

#### Statistical analysis

Analyses were performed using the Two-Sample MR R package (version 4.1.0) with a significance level of α = 0.05. For analyses involving multiple microbial taxa, p-values were adjusted using the False Discovery Rate (FDR) method. An FDR-adjusted q-value < 0.05 was considered statistically significant. FDR-adjusted q-values are presented in Supplementary Table S2.

## Results

### Causal effect of genetically predicted TTK inhibition on breast cancer risk

The authors identified two robust SNPs (rs7745127 and rs11960958) from the TTK eQTL data as instrumental variables (Supplementary Table S3). The F-statistics for these SNPs were 37.1 and 27.5, respectively, indicating a low risk of weak instrument bias. The two-sample MR analysis revealed a significant protective effect of genetically predicted TTK inhibition on BC risk. The Inverse-Variance Weighted (IVW) method estimated an Odds Ratio (OR) of 0.667 (95 % CI 0.543‒0.819), which remained significant after FDR correction (*p* < 0.001; FDR-adjusted *p* < 0.001) (Table 1). The Weighted Median analysis showed a consistent direction of effect (OR = 0.692, 95 % CI 0.515‒0.930, *p* = 0.015), providing additional support despite the limited number of SNPs (Table 1). No evidence of heterogeneity was found between the SNPs (I² = 0 %, Cochran’s *Q*-test *p* = 0.834).

**Table 1:** Estimates of the causal effect of genetically predicted TTK inhibition on breast cancer.

Exposure	Outcome	Method	NSNP	MR OR (95 % CI)	MR p-value	Heterogeneity p-value
BOS172722	Breast cancer	IVW (Fixed Effects)	2	0.667 (0.543‒0.819)	<0.001 (FDR <0.001)	0.834
BOS172722	Breast cancer	Weighted Median	2	0.692 (0.515‒0.930)	0.015	NA

MR, Mendelian Randomization; NSNP, Number of SNPs; OR, Odds Ratio; 95 % CI, 95 % Confidence Interval; FDR, False Discovery Rate; IVW, Inverse Variance Weighted. Weighted median analysis is included as a sensitivity check, though interpretation is limited by the low number of SNPs.

### Exploratory analysis of TTK inhibition and gut microbiota on breast cancer

The authors next performed a broad, exploratory analysis to assess the causal effects of TTK inhibition on 211 gut microbial taxa and the corresponding effects of these taxa on BC risk. In the initial uncorrected analyses, genetically predicted TTK inhibition was nominally associated with 20 microbial taxa (*p* < 0.05), while 6 taxa showed a nominal association with BC risk (Supplementary Tables S4 and S5). The forest plot in Supplementary Figure S1 visualizes these nominal associations. However, after applying FDR correction for multiple testing, no associations between TTK inhibition and any of the 211 gut microbial taxa, nor between any of the taxa and BC risk, remained statistically significant (all q-values >0.1; Supplementary Table S2).

### Exploratory mediation analysis of a nominally significant taxon

**Fig. 2:**
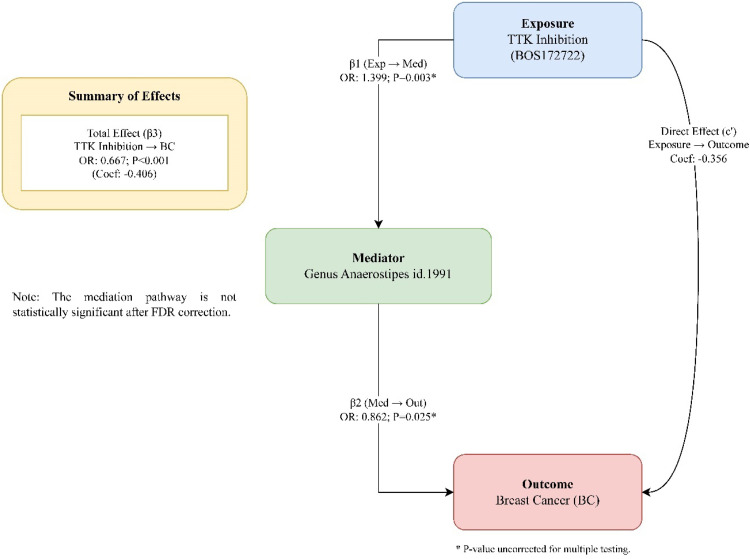
Potential causal associations and exploratory mediation pathway. The diagram summarizes the main MR finding of TTK inhibition on breast cancer risk (Total Effect, β3) and the exploratory mediation pathway via *Genus Anaerostipes* id.1991. Effects are shown with Odds Ratios (OR) and corresponding p-values. Coef.: Coefficient. *p-value is uncorrected for multiple testing. **p-value is FDR-adjusted. Note: The mediation pathway is not statistically significant after FDR correction and is presented for exploratory hypothesis generation only.

Although no microbial taxa showed a statistically significant association with both TTK inhibition and BC after FDR correction, the authors conducted a purely exploratory analysis on *Genus Anaerostipes* id.1991, which showed the strongest nominal signals in both steps (TTK inhibition → *Anaerostipes*: OR = 1.399, 95 % CI 1.117‒1.753, uncorrected *p* = 0.003; *Anaerostipes* → BC: OR = 0.862, 95 % CI 0.757‒0.982, uncorrected *p* = 0.025). This analysis is intended for hypothesis-generation only, given the lack of statistical significance of its constituent pathways after multiple testing correction. To strengthen the proposed directionality, the authors performed a reverse MR analysis, which showed no causal effect of BC on *Genus Anaerostipes* id.1991 (OR = 1.002, 95 % CI 0.970‒1.035, *p* = 0.920) (Table 2). This analysis suggested a potential partial mediation pathway; however, this finding is not statistically significant and is intended solely for hypothesis generation (Supplementary Table S6; Fig. 2).

**Table 2:** Estimates of the causal effect of breast cancer on *Genus Anaerostipes* id.1991.

Exposure	Outcome	Method	NSNP	MR OR (95 %CI)	MR p-value
BC	*Genus Anaerostipes* id.1991	Inverse variance weighted	85	1.002 (0.970‒1.035)	0.920
BC	*Genus Anaerostipes* id.1991	MR Egger	85	1.008 (0.934‒1.087)	0.843
BC	*Genus Anaerostipes* id.1991	Simple mode	85	0.928 (0.833‒1.033)	0.176
BC	*Genus Anaerostipes* id.1991	Weighted median	85	0.998 (0.947‒1.050)	0.925
BC	*Genus Anaerostipes* id.1991	Weighted mode	85	0.999 (0.938‒1.064)	0.972

BC, Breast Cancer; MR, Mendelian Randomization; NSNP, Number of SNPs; OR, Odds Ratio; 95 % CI, 95 % Confidence Interval. For MR analysis, horizontal pleiotropy was considered significant if *p* < 0.05. MR-PRESSO test for pleiotropy was not significant (*p* = 0.902). The MR-Egger intercept p-value was 0.863, indicating no directional pleiotropy.

## Discussion

In this two-step, two-sample drug-target MR study, the primary and most robust finding is that genetically predicted TTK inhibition is causally associated with a reduced risk of BC. This result withstood stringent multiple testing correction and provides genetic validation for TTK as a promising therapeutic target. The secondary, exploratory analysis investigated the potential mediating role of the gut microbiome. Although the authors identified nominal associations between TTK inhibition and several gut microbes, and between certain microbes and BC risk, none of this survived FDR correction. Consequently, the mediation analysis, which focused on the nominally significant Genus *Anaerostipes*, should be interpreted as purely hypothesis-generating rather than confirmatory.

The primary finding validates the therapeutic potential of TTK inhibitors in BC using human genetic data. This aligns with preclinical studies showing that TTK inhibition induces apoptosis and cell cycle arrest in BC cell lines.^14^^,^^44^ While several TTK inhibitors are in clinical trials,^45^^,^^46^ the present study offers population-level genetic evidence supporting their efficacy. TTK inhibition has also shown promise in overcoming drug and radio-resistance in BC,^47^ highlighting its potential as a broad therapeutic strategy that warrants further clinical investigation.

The bidirectional relationship between the gut microbiota and BC is an area of intense research.^48^, ^49^, ^50^ Prior studies have linked specific microbial taxa, such as those from the *Ruminococcaceae* and *Lachnospiraceae* families, to BC risk, sometimes with conflicting results depending on the study population and BC subtype.^18^^,^^51^^,^^52^ The present study initially identified several such taxa with nominal associations, but the lack of significance after FDR correction underscores the challenge of identifying robust effects in microbe-wide association studies and the importance of stringent statistical correction. This suggests that the influence of individual taxa may be modest or context-dependent, requiring larger sample sizes for definitive detection.

Based on uncorrected p-values, *Genus Anaerostipes* emerged as a candidate for exploratory mediation analysis. While this finding is not statistically robust, it allows for hypothesis generation. For instance, *Anaerostipes* is a known producer of butyrate, a Short-Chain Fatty Acid (SCFA) with documented anti-proliferative and anti-inflammatory properties in vitro, including in BC cell lines.^24^^,^^53^, ^54^, ^55^ Whether TTK inhibition could modulate such bacteria and thereby influence BC progression via metabolites like SCFAs remains a speculative but intriguing hypothesis for future mechanistic studies. However, it is critical to reiterate that the present data do not provide sufficient statistical evidence to support this specific mediatory pathway.

The present study's strengths include the use of a two-sample MR design to mitigate confounding and reverse causation, supported by high F-statistics for these instruments. However, it has significant limitations. First, the analysis of genetically predicted TTK inhibition relied on only two instrumental SNPs. Although their F-statistics were well above the conventional threshold of 10, indicating sufficient strength against weak instrument bias, this small number severely limits the scope of sensitivity analyses. Specifically, it precluded the use of methods like MR-Egger regression and MR-PRESSO to robustly assess horizontal pleiotropy. Although the authors performed a Weighted Median analysis (Table 1), which showed a consistent direction of effect, the low number of SNPs limits the reliability of these sensitivity checks. Consequently, the authors cannot definitively rule out the possibility that the observed effect is biased by pleiotropic pathways, and these findings should be interpreted as suggestive rather than definitive, requiring replication with more instruments in future studies. Secondly, the exclusive use of European-ancestry data limits the generalizability of the present findings. Previous studies have highlighted significant disparities in breast cancer subtypes and gut microbiome composition across different ethnicities.^56^^,^^57^ Therefore, these findings may not be applicable to non-European populations, and multi-ancestry replication studies are essential. Thirdly, the present study models the lifetime effect of genetic predisposition, which may not directly correspond to the short-term pharmacological effects of TTK inhibitors in a clinical setting. Finally, the extensive multiple testing in the microbiota analysis underscores that the nominal findings should be treated with extreme caution and primarily as a basis for future, more targeted research.

## Conclusion

In conclusion, the MR analysis provides robust genetic evidence for a causal, protective effect of TTK inhibition on the risk of breast cancer. This reinforces the potential of TTK as a therapeutic target. The exploratory investigation into the mediating role of the gut microbiome, specifically *Genus Anaerostipes*, did not yield statistically significant results after correction for multiple testing. Therefore, while the gut-cancer axis remains a promising field, the specific role of *Anaerostipes* as a mediator in this context is not supported by the present data and requires further validation in larger, adequately powered studies.

## Declarations

Ethics approval and consent to participate: Not applicable.

Consent for publication: Not applicable.

Availability of data and material: The datasets generated and analyzed during the current study are available in the supplementary materials.

## Funding

This study was supported by National High-Level Hospital Clinical Research Funding (2022‐PUMCH‐B‐038), 10.13039/501100002858China Postdoctoral Science Foundation (2023M730328), and 10.13039/501100002858China Postdoctoral Science Foundation (2023T160063).

## Data availability

The datasets generated and/or analyzed during the current study are available from the corresponding author upon reasonable request.

## CRediT authorship contribution statement

**Jia Liu:** Conceptualization, Writing – original draft. **Mohan Liu:** Data curation, Formal analysis. **Ying Xu:** Software, Validation. **Zihao Wang:** Formal analysis, Funding acquisition. **Xingtong Zhou:** Conceptualization, Writing – original draft, Writing – review & editing, Project administration. **Qiang Sun:** Conceptualization, Funding acquisition, Writing – review & editing.

## Conflicts of interest

The authors declare no conflicts of interest.

## Appendix Supplementary materials

Image, application 1

## Footnotes

Supplementary material associated with this article can be found, in the online version, at doi:10.1016/j.clinsp.2026.100961.
