# Supplementary material for: Genetically predicted TTK inhibition and its association with reduced breast cancer risk: a two-step Mendelian randomization study of potential gut microbiome mediation
Source: Clinics (Sao Paulo). 2026 May 1;81:100961. doi: 10.1016/j.clinsp.2026.100961 (PMC13147426; doi:10.1016/j.clinsp.2026.100961)

CLINICS-D-24-01417_Supplementary Material

**Supplementary Tables**

**Supplementary Table S1** Detailed information on datasets used in the Mendelian randomization analysis.

| **Data source** | **Phenotype** | **Sample size** | **Cases** | **Population** | **Adjustment** |
| --- | --- | --- | --- | --- | --- |
| eqtl-a-ENSG00000112742 | TTK | 31684 | ‒ | European | Males and Females |
| ebi-a-GCST90018799 | Breast cancer | 257730 | 17389 | European | ‒ |
| IEU Open GWAS project | Gut microbiota | 14306 | ‒ | European | ‒ |

**Supplementary Table S2** FDR-adjusted q-values for MR analyses of TTK inhibition on gut microbiota and gut microbiota on BC risk.

| **Analysis Step** | **Exposure** | **Outcome (Microbiota Taxon)** | **Original p-value** | **FDR q-value** |
| --- | --- | --- | --- | --- |
| **Step 1: TTK Inhibition on Gut Microbiota (Nominally significant taxa)** | | | | |
| TTK Inhibition -> Microbiota | BOS172722 (TTK) | Class Clostridia id.1859 | 0.015 | 0.602702027 |
| TTK Inhibition -> Microbiota | BOS172722 (TTK) | Class Verrucomicrobiae id.4029 | 0.031 | 0.602702027 |
| TTK Inhibition -> Microbiota | BOS172722 (TTK) | Family Bacteroidales S24 7group id.11173 | 0.048 | 0.602702027 |
| TTK Inhibition -> Microbiota | BOS172722 (TTK) | Family Oxalobacteraceae id.2966 | 0.042 | 0.602702027 |
| TTK Inhibition -> Microbiota | BOS172722 (TTK) | Family Verrucomicrobiaceae id.4036 | 0.031 | 0.602702027 |
| TTK Inhibition -> Microbiota | BOS172722 (TTK) | Genus Akkermansia id.4037 | 0.033 | 0.602702027 |
| TTK Inhibition -> Microbiota | BOS172722 (TTK) | Genus Anaerostipes id.1991 | 0.003 | 0.370184706 |
| TTK Inhibition -> Microbiota | BOS172722 (TTK) | Genus Coprococcus3 id.11303 | 0.026 | 0.602702027 |
| TTK Inhibition -> Microbiota | BOS172722 (TTK) | Genus Dorea id.1997 | 0.002 | 0.370184706 |
| TTK Inhibition -> Microbiota | BOS172722 (TTK) | Genus Eisenbergiella id.11304 | 0.013 | 0.602702027 |
| TTK Inhibition -> Microbiota | BOS172722 (TTK) | Genus Eubacteriumcoprostanoligenes group id.11375 | 0.003 | 0.370184706 |
| TTK Inhibition -> Microbiota | BOS172722 (TTK) | Genus Eubacteriumrectale group id.14374 | 0.046 | 0.602702027 |
| TTK Inhibition -> Microbiota | BOS172722 (TTK) | Genus Senegalimassilia id.11160 | 0.002 | 0.370184706 |
| TTK Inhibition -> Microbiota | BOS172722 (TTK) | Genus Subdoligranulum id.2070 | 0.016 | 0.602702027 |
| TTK Inhibition -> Microbiota | BOS172722 (TTK) | Unknown genus id.1000005479 | 0.048 | 0.602702027 |
| TTK Inhibition -> Microbiota | BOS172722 (TTK) | Unknown genus id.959 | 0.024 | 0.602702027 |
| TTK Inhibition -> Microbiota | BOS172722 (TTK) | Order Clostridiales id.1863 | 0.016 | 0.602702027 |
| TTK Inhibition -> Microbiota | BOS172722 (TTK) | Order Verrucomicrobiales id.4030 | 0.031 | 0.602702027 |
| TTK Inhibition -> Microbiota | BOS172722 (TTK) | Phylum Firmicutes id.1672 | 0.007 | 0.525232919 |
| TTK Inhibition -> Microbiota | BOS172722 (TTK) | Phylum Proteobacteria id.2375 | 0.037 | 0.602702027 |
| **Step 2: Gut Microbiota on BC Risk (Nominally significant taxa)** | | | | |
| Microbiota -> BC | Family Family XI id.1936 | Breast cancer | 0.042 | >0.1 |
| Microbiota -> BC | Genus Anaerostipes id.1991 | Breast cancer | 0.025 | >0.1 |
| Microbiota -> BC | Genus Lachnospiraceae NK4A136 group id.11319 | Breast cancer | 0.007 | >0.1 |
| Microbiota -> BC | Genus Ruminiclostridium5 id.11355 | Breast cancer | 0.018 | >0.1 |
| Microbiota -> BC | Genus Ruminococcaceae UCG014 id.11371 | Breast cancer | 0.031 | >0.1 |
| Microbiota -> BC | Phylum Actinobacteria id.400 | Breast cancer | 0.020 | >0.1 |

FDR, False Discovery Rate. All analyses were corrected for 211 microbial taxa comparisons.

**Supplementary Table S3** Characteristics of instrumental variables for TTK inhibition.

| **SNP** | **Outcome** | **CHR** | **POS** | **EA/OA** | **EAF** | **β** | **SE** | **p** | **F-statistic** | **MAF** |
| --- | --- | --- | --- | --- | --- | --- | --- | --- | --- | --- |
| rs7745127 | Breast cancer | 6 | 80760668 | C/T | 0.589 | -0.028 | 0.010 | 0.006 | 37.109 | 0.411 |
| rs11960958 | Breast cancer | 6 | 81199635 | G/A | 0.350 | -0.029 | 0.011 | 0.007 | 27.529 | 0.350 |

SNP, Single Nucleotide Polymorphism; CHR, Chromosome; POS, Position; EA, Effect Allele; OA, Other Allele; EAF, Effect Allele Frequency; β, Ceta Coefficient; SE, Standard Error; p, p-value; F-statistic, a measure of instrument strength; MAF, Minor Allele Frequency.

**Supplementary Table S4** Estimates of nominal causal effect of genetically predicted TTK inhibition on gut microbiota.

| **Exposure** | **Outcome** | **NSNP** | **MR OR (95%CI)** | **MR p-value (uncorrected)** | **Heterogeneity I² (%)** | **Heterogeneity p-value** |
| --- | --- | --- | --- | --- | --- | --- |
| BOS172722 | Class Clostridia id.1859 | 2 | 1.308 (1.054‒1.624) | 0.015 | 0 | 0.355 |
| BOS172722 | Class Verrucomicrobiae id.4029 | 2 | 0.744 (0.568‒0.973) | 0.031 | 0 | 0.403 |
| BOS172722 | Family Bacteroidales S24 7group id.11173 | 2 | 0.719 (0.518‒0.998) | 0.048 | 0 | 0.888 |
| BOS172722 | Family Oxalobacteraceae id.2966 | 2 | 0.656 (0.437‒0.985) | 0.042 | 0 | 0.853 |
| BOS172722 | Family Verrucomicrobiaceae id.4036 | 2 | 0.744 (0.569‒0.973) | 0.031 | 0 | 0.408 |
| BOS172722 | Genus Akkermansia id.4037 | 2 | 0.746 (0.570‒0.976) | 0.033 | 0 | 0.411 |
| BOS172722 | Genus Anaerostipes id.1991 | 2 | 1.399 (1.117‒1.753) | 0.003 | 0 | 0.406 |
| BOS172722 | Genus Coprococcus3 id.11303 | 2 | 1.302 (1.032‒1.644) | 0.026 | 0 | 0.463 |
| BOS172722 | Genus Dorea id.1997 | 2 | 1.420 (1.139‒1.771) | 0.002 | 0 | 0.466 |
| BOS172722 | Genus Eisenbergiella id.11304 | 2 | 0.609 (0.412‒0.900) | 0.013 | 0 | 0.446 |
| BOS172722 | Genus Eubacteriumcoprostanoligenes group id.11375 | 2 | 1.397 (1.119‒1.744) | 0.003 | 0 | 0.830 |
| BOS172722 | Genus Eubacteriumrectale group id.14374 | 2 | 1.254 (1.004‒1.566) | 0.046 | 0 | 0.696 |
| BOS172722 | Genus Senegalimassilia id.11160 | 2 | 1.744 (1.228‒2.479) | 0.002 | 0 | 0.454 |
| BOS172722 | Genus Subdoligranulum id.2070 | 2 | 1.311 (1.051‒1.635) | 0.016 | 0 | 0.544 |
| BOS172722 | Unknown genus id.1000005479 | 2 | 0.719 (0.518‒0.998) | 0.048 | 0 | 0.888 |
| BOS172722 | Unknown genus id.959 | 2 | 0.617 (0.406‒0.940) | 0.024 | 0 | 0.638 |
| BOS172722 | Order Clostridiales id.1863 | 2 | 1.306 (1.052‒1.621) | 0.016 | 0 | 0.351 |
| BOS172722 | Order Verrucomicrobiales id.4030 | 2 | 0.744 (0.568‒0.973) | 0.031 | 0 | 0.403 |
| BOS172722 | Phylum Firmicutes id.1672 | 2 | 1.349 (1.087‒1.675) | 0.007 | 0 | 0.724 |
| BOS172722 | Phylum Proteobacteria id.2375 | 2 | 0.794 (0.640‒0.987) | 0.037 | 0 | 0.543 |

MR, Mendelian Randomization; NSNP, Number of SNPs; OR, Odds Ratio; 95%CI, 95% Confidence Interval. p-values presented for gut microbiota associations are uncorrected; all FDR-adjusted q-values for these associations were > 0.1 (see Supplementary Table S2).

**Supplementary Table S5** Estimates of nominal causal effect of gut microbiota on breast cancer.

| **Exposure** | **Outcome** | **NSNP** | **MR OR (95%CI)** | **MR p-value (uncorrected)** | **Pleiotropy (Egger Intercept p-value)** |
| --- | --- | --- | --- | --- | --- |
| Family Family XI id.1936 | Breast cancer | 4 | 1.105 (1.004‒1.215) | 0.042 | 0.246 |
| Genus Anaerostipes id.1991 | Breast cancer | 12 | 0.862 (0.757‒0.982) | 0.025 | 0.482 |
| Genus Lachnospiraceae NK4A136 group id.11319 | Breast cancer | 11 | 0.876 (0.795‒0.965) | 0.007 | 0.829 |
| Genus Ruminiclostridium5 id.11355 | Breast cancer | 8 | 0.853 (0.748‒0.973) | 0.018 | 0.588 |
| Genus Ruminococcaceae UCG014 id.11371 | Breast cancer | 7 | 0.846 (0.727‒0.985) | 0.031 | 0.472 |
| Phylum Actinobacteria id.400 | Breast cancer | 14 | 1.148 (1.022‒1.289) | 0.020 | 0.278 |

MR, Mendelian Randomization; NSNP, Number of SNPs; OR, Odds Ratio; 95%CI, 95% Confidence Interval. p-values presented for gut microbiota associations are uncorrected; all FDR-adjusted q-values for these associations were >0.1 (see Supplementary Table S2).

**Supplementary Table S6** Exploratory mediation effect of genetically predicted TTK inhibition on breast cancer through *Genus Anaerostipes* id.1991.

| **Mediation Path (based on uncorrected p-values)** | **Mediation Effect** | **Direct Effect** | **Total Effect** | **Proportion Mediated (%) (95% CI)** |
| --- | --- | --- | --- | --- |
| *Genus Anaerostipes* id.1991 | -0.050 | -0.356 | -0.406 | 12.326% (6.615% ‒ 18.037%) |

Effects are presented as coefficients. This analysis is exploratory and based on nominally significant associations that did not survive correction for multiple testing.

**Supplementary Figure S1** Forest plot of the effect of BOS172722 on gut microbiota and the effect of gut microbiota on BC. OR, Odds Ratio; 95% CI, 95% Confidence Interval. Note: These associations are nominally significant (uncorrected p<0.05) but did not withstand FDR correction.


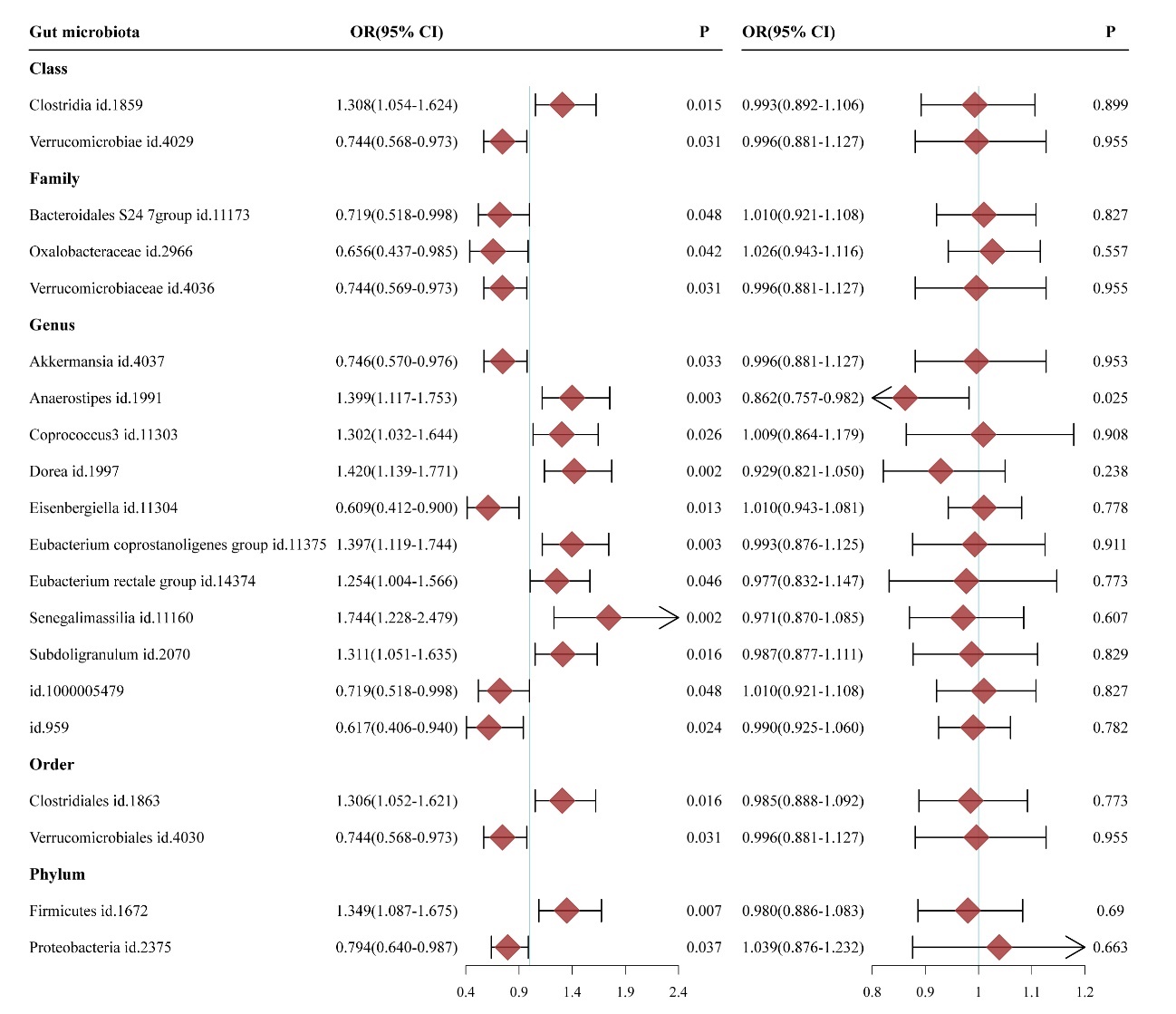

Supplement: Supplementary file 1 [file mmc1.docx]
